# Bulk and Surface Acoustic Wave Sensor Arrays for Multi-Analyte Detection: A Review

**DOI:** 10.3390/s19245382

**Published:** 2019-12-06

**Authors:** Kerstin Länge

**Affiliations:** Institute of Microstructure Technology, Karlsruhe Institute of Technology, Hermann-von-Helmholtz-Platz 1, 76344 Eggenstein-Leopoldshafen, Germany; kerstin.laenge@kit.edu; Tel.: +49-721-608-22673

**Keywords:** bulk acoustic wave, surface acoustic wave, quartz crystal microbalance, film bulk acoustic resonator, sensor array, chemical sensor, biosensor, electronic nose, electronic tongue

## Abstract

Bulk acoustic wave (BAW) and surface acoustic wave (SAW) sensor devices have successfully been used in a wide variety of gas sensing, liquid sensing, and biosensing applications. Devices include BAW sensors using thickness shear modes and SAW sensors using Rayleigh waves or horizontally polarized shear waves (HPSWs). Analyte specificity and selectivity of the sensors are determined by the sensor coatings. If a group of analytes is to be detected or if only selective coatings (i.e., coatings responding to more than one analyte) are available, the use of multi-sensor arrays is advantageous, as the evaluation of the resulting signal patterns allows qualitative and quantitative characterization of the sample. Virtual sensor arrays utilize only one sensor but combine it with enhanced signal evaluation methods or preceding sample separation, which results in similar results as obtained with multi-sensor arrays. Both array types have shown to be promising with regard to system integration and low costs. This review discusses principles and design considerations for acoustic multi-sensor and virtual sensor arrays and outlines the use of these arrays in multi-analyte detection applications, focusing mainly on developments of the past decade.

## 1. Introduction

Sensors have become indispensable in chemical and biological analytics. If samples contain more than one analyte of interest, sensor arrays are advantageous because they enable the detection of more than one analyte in a single measurement run, particularly if analyte-specific coatings are available. If only selective coatings are at hand, sensor arrays are even a must for the reliable detection of a single analyte. The combination of suitable selective coatings and evaluation algorithms, including pattern recognition methods, also allows qualitative and quantitative determination of several analytes in mixtures. While the sensor coating determines selectivity or specificity of an assay, the sensor device (transducer) determines the sensitivity of the assay. Today, a large number of sensors are available, mainly utilizing electrochemical, optical, or acoustic signal transduction. Sensors with acoustic signal transduction detect, among others, the mass of an analyte, i.e., an inherent property of every analyte, which makes them universal in use. The sensor devices can be manufactured very small down to submillimeter dimensions, which enables the design of correspondingly small arrays. Furthermore, acoustic transducers can easily be integrated in wireless communication systems [1,2,3,4,5].

Acoustic sensors and biosensors offer label-free, fast, sensitive, and low-cost detection of analytes in both gaseous and liquid samples. A large variety of acoustic sensor devices is available using mainly bulk or surface acoustic waves. The devices have in common that they utilize both the piezoelectric and the inverse piezoelectric effect, i.e., their operation principle includes interconversion and detection of electrical energies and acoustic (i.e., mechanical) waves. The velocity of the acoustic wave and, hence, the sensor signal response is influenced, among others, by mass changes on the device surface [1,6,7]. The evaluation of chemical sensor signal responses is mainly based on the signal shifts and the resulting signal patterns. Sometimes the signal development over time is also considered, including compensation of potential sensor drifts [8,9,10,11]. Biosensor signals obtained by diffusion-limited analyte binding on the surface are linear with the slope being proportional to the analyte concentration. Evaluation of signals resulting from kinetically controlled analyte binding on the surface is mainly used for the determination of kinetic and thermodynamic constants of the surface reaction [12,13]. 

Chemical sensor arrays with selective coatings for the characterization of complex gaseous mixtures are also called “electronic noses” (e-noses), while their counterparts for liquid samples are known as “electronic tongues” (e-tongues) [14,15]. A variety of coating materials for acoustic chemical sensor arrays has been developed, with polymer-based coatings representing the largest group. Even the pure polymers offer a wide range of coatings because of the multitude of functional groups and structures available. Additionally, molecularly imprinted polymers (MIPs) have been developed to obtain higher selectivities. Highly selective MIP-coated sensors have also been referred to as “chemosensors” as a counterpart to the analyte-specific biosensors (see below) [16,17,18,19,20]. Other organic coating materials have been derived from self-assembled monolayers (e.g., silanes), macrocycles (e.g., calixarenes, cyclodextrins, phthalocyanines, and porphyrins), and organic salts (ionic liquids and GUMBOS (group of uniform materials based on organic salts)) [16,21,22,23,24,25]. Inorganic coating materials include metal oxides and carbonaceous materials, such as graphene or graphene oxide, carbon nanotubes (CNTs), multi-walled CNTs (MWCNTs), and diamond nanoparticles [16,26,27,28]. Recent developments regarding the enhancement of the selectivity of chemical sensors take advantage of biological molecules as coatings, such as DNA forming loops, peptides, and proteins (e.g., odorant-binding proteins) [29,30,31,32,33]. Biosensors represent the combination of a transducer with an analyte-specific biorecognition element. They can be used as single components for specific detection of the corresponding analytes. However, biosensor arrays would be convenient for a higher throughput if several analytes are to be determined. Coatings for acoustic biosensor arrays have been predominated by antibodies as specific capture molecules for the corresponding analytes. The use of single-stranded DNA to capture corresponding DNA strands has also been reported [34,35,36]. 

Acoustic sensor array applications include quantitative determination of sample compounds and qualitative determination of compound patterns, e.g., to determine health profiles or food quality, where it is not necessarily required to know exactly the contributing components or their concentrations. Acoustic e-noses have been used for the detection of volatile organic compounds (VOCs), chemical warfare agents (CWAs), volatile biomarkers, and odors. Correspondingly, gaseous samples have consisted of indoor, outdoor, or artificial air, breath, and headspace of liquid or solid samples, such as wastewater, food, and plants [25,37,38,39,40,41,42,43,44]. Further applications include the use as detector for gas chromatography (GC) instead of mass spectrometry (MS) [45,46] and the use as sensor node in sensor networks [47]. In contrast to that, the use of acoustic sensor arrays as e-tongues or biosensor arrays for liquid samples has been much less common. Applications include the detection of contaminants in water and of biomarkers in body fluids, e.g., for diagnostics [35,48,49,50,51].

In the following, the components of acoustic sensor arrays are discussed and an overview of commercially available acoustic sensor arrays and e-noses is given. After that, applications of multi-sensor and virtual sensor arrays for multi-analyte detection are summarized, with the main focus on research work of the past decade.

## 2. Configuration of Acoustic Sensor Arrays

### 2.1. Acoustic Sensor Devices

**Figure 1:**
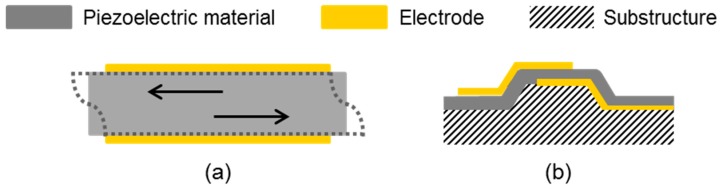
Schematics of bulk acoustic wave (BAW) devices: (**a**) quartz crystal microbalances (QCMs) and (**b**) film bulk acoustic resonators (FBARs).

The oldest and still most commonly used acoustic sensor devices are quartz crystal microbalances (QCMs), also known as quartz microbalances (QMBs), which belong to bulk acoustic wave (BAW) devices (Figure 1). As the phrase suggests, they are made of quartz, where mostly the AT cut is used. The typical setup is depicted in Figure 1a. It shows a quartz disk with electrodes mounted on both surfaces generating thickness shear modes with common frequencies in the range of 5 to 50 MHz. QCM sensors have been used for both gas and liquid sensing, including biosensing applications. The resonance frequency, which is linked to the deposited mass, is the main parameter recorded during the measurements. Some instruments (see Table 1 in Section 2.4) additionally enable the recording of the dissipation, allowing conclusions about the viscoelasticity of the deposited layer to be drawn [6,52,53].

Though QCMs are well known and widespread, other devices using higher frequencies are desired, as they may promise higher mass sensitivities. QCM sensors at higher frequencies are producible (see Table 1 in Section 2.4); but the devices get thinner with increasing frequencies, making them fragile and more difficult to handle. Other approaches to enhance the QCM sensor performance aim at electrodeless or wireless-electrodeless configurations. Thin piezoelectric devices may also support other wave types, such as acoustic plate modes (APM) and flexural plate waves (FPW). APM devices, where the waves are guided within the device by reflection from the surfaces, provide operation frequencies in the range of 20–200 MHz. However, as usually several plate modes are excited and differ only slightly in the frequency, expensive evaluation electronics are required. The frequency range of FPW devices is only in the range of 5–20 MHz, and the devices are still fragile, which therefore means no advantage compared to QCMs [54,55,56,57].

More recent developments in BAW devices comprise film bulk acoustic resonators (FBARs), also known as thin film bulk acoustic resonators (TFBARs). They are mostly made of aluminum nitride (AlN) or zinc oxide (ZnO) thin films. A general setup of FBARs is depicted in Figure 1b. The resonator films are solidly mounted on a support structure, resulting in one of three FBAR types, i.e., back trench, Bragg acoustic mirror, or air-bag type. In principle, the fabrication of FBARs is compatible to complementary metal oxide semiconductor (CMOS) technology (unless ZnO is used). FBARs can be operated in longitudinal mode or in thickness shear mode, where the latter is to be preferred in liquids to minimize energy loss. FBARs allow operation frequencies ranging from sub-GHz to tens of GHz. The resonators have been applied in gas sensing and biosensing applications [58,59,60].

**Figure 2:**
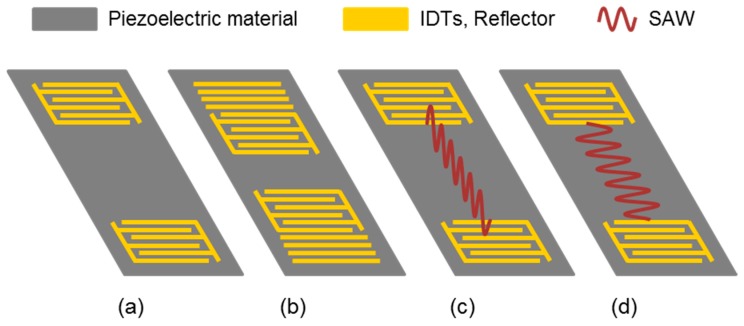
Schematics of surface acoustic wave (SAW) devices: (**a**) Delay line device; (**b**) resonator device; (**c**) Rayleigh wave; and (**d**) horizontally polarized shear wave (HPSW). IDTs: interdigital transducers.

The other large group of acoustic sensors is represented by surface acoustic wave (SAW) devices (Figure 2), which allow operation frequencies in the range of a few MHz to a few GHz. The wave on the surface of the piezoelectric material is excited and received by interdigital transducers (IDTs), a specific type of electrode. The IDTs are mainly designed in two configurations leading to either delay line or resonator devices (Figure 2a,b). The spacing between the input and output IDTs in the delay line configuration causes a time delay between input and output signals, which is why preferably phase and amplitude shifts of the SAW are recorded, requiring comparatively complex electronics. In the two-port resonator configuration (Figure 2b), input and output IDTs are closer together and surrounded by reflective fingers. One-port resonators have only one IDT in the middle of the device, with reflective fingers on both sides. The reflective structures of SAW resonators lead to very distinct and sharp resonance frequencies, which can easily be collected by simple and economical electronic setups, such as oscillators. SAW devices are mainly produced by lithography and subsequent metal deposition, which can be carried out as mass production processes [53,55,61]. 

The most commonly used piezoelectric materials for SAW sensors are quartz, lithium niobate (LiNbO_3_), and lithium tantalate (LiTaO_3_). Depending on piezoelectric material and crystal cut, different wave types are obtained. Rayleigh waves are characterized by particle displacement perpendicular to the surface (Figure 2c). SAW sensors for gas sensing applications are commonly based on Rayleigh wave devices. If they are used in liquid media, however, an immense attenuation loss is observed because the particle displacement perpendicular to the surface generates compression waves radiating into the liquid. Therefore, SAW devices to be used in liquids require waves supporting shear horizontal particle displacements, such as horizontally polarized shear waves (HPSWs) (Figure 2d). This includes Love waves and surface transverse waves (STWs), where the wave is guided on top of the device in a thin guiding layer (Love waves) or by a metal strip grating (STWs). While Rayleigh wave devices are restricted to gas sensing applications, SAW devices supporting HPSWs can be used for both liquid and gas sensing applications [55,56,60,61]. 

### 2.2. Measuring with Acoustic Sensors

To obtain high-performance sensor setups, the respective application must be considered and both sensor devices and sensor coatings have to be carefully selected and, if possible, adapted. As mentioned in Section 2.1, liquid sample applications with acoustic sensors require waves moving in parallel to the surface, without particle displacement perpendicular to the surface, such as the (thickness) shear modes of QCMs or FBARs and the HPSWs of SAW devices. In contrast to that, measurements with gaseous samples can be performed with both acoustic waves moving in parallel and acoustic waves moving perpendicular to the surface. The appropriate wave type would be obtained by choosing the appropriate piezoelectric material and crystal cut. If possible, this choice should also consider the temperature stability of the crystals. AT-cut quartz, for instance, provides comparatively stable frequencies over a wide temperature change (variation 0–25 ppm over −50 °C to 100 °C). Materials used for SAW devices, however, such as LiNbO_3_ and LiTaO_3_, show higher frequency changes associated with temperature changes. This limitation can be overcome by additional quartz (SiO_2_) layers reducing this effect or by providing a suitable external thermostatic control in the final measurement setup. The latter would also be useful to reduce temperature effects on the kinetics of analyte adsorption or affinity binding on the sensor surface [5,53,55,62,63].

Acoustic sensors are generally regarded as mass-sensitive sensors. In the gravimetric regime, increased frequency shifts are obtained by mass loading when acoustic devices with higher operation frequencies are used. Therefore, newer developments include high-frequency devices, such as FBARs. However, as long as the higher operation frequencies are associated with higher noise, a higher mass-sensitivity is not necessarily achieved [5,51,54]. Another parameter influencing the sensor response is the composition of the sensing layer with regard to viscoelasticity, which is connected with the penetration depth and, therefore, the sensing zone of the acoustic wave. Changes in viscoelasticity may add to the effect of mass loading, resulting in increased sensor responses, as observed, for instance, for SAW chemical sensors with specific polymer coatings. However, viscoelasticity change and mass loading may also counteract each other, leading to reduced sensor responses, as observed, for instance, for SAW biosensors with comparatively thick sensing layers. This is associated with the reduction of the penetration depth of an acoustic wave into the medium by higher device frequencies. In the worst case, a sensing layer is developed with excellent analyte-binding properties, but if the layer thickness exceeds the penetration depth of the acoustic wave, binding events too far away from the device surface could not be detected, leading to reduced signal responses. Using thin, two-dimensional sensing layers allowing the analyte to bind only on top of the layer can minimize these disadvantageous effects resulting from viscoelasticity changes [64,65,66,67,68,69].

Apart from mass loading and viscoelasticity changes, acoustic sensor signals may also be affected by changes in the electrical environment influencing the electromechanical coupling. This is particularly an issue for liquid sample applications. For sensors based on QCMs and on SAW delay lines, this effect has effectively been eliminated by introducing metal coatings, which shield the acoustic wave from the differential electrical influences of the liquids, such as conductivity. When working with SAW resonators, however, changes in the electrical environment still have a high impact on the sensor response. One way to overcome this problem is to adapt the carrier medium transporting the liquid samples to the sample background in a way that the electrical differences are minimized. Newer approaches include the combination of SAW resonators with electrical sensors resulting in a dual signal response, which would allow an improved characterization of the individual sensor responses [54,70,71,72,73,74].

The coating of acoustic sensors has to meet both the requirements based on the acoustic transduction principle, as mentioned above, and the requirements arising from the sensor application. Thick layers may be advantageous to capture many analyte molecules for a high sensor response, but the thickness must not reduce the sensor response because of counteracting effects. Nanostructures to increase the layer capacity have also been reported. However, for acoustic transducers the structure dimensions cannot be chosen arbitrarily because structure sizes in the range of the acoustic wavelength could lead to scattering effects and, hence, to energy loss. While biosensor coatings may be highly specific for an individual analyte, coatings for chemical sensors are typically selective for a group of chemically similar analyte molecules. On the other hand, chemical sensors can often be used multiple times when surface regeneration is possible by flushing with clean air, maybe at a slightly elevated temperature. Regeneration is not that easily possible for biosensors binding the analyte with high affinity. However, biosensors have mainly been developed for clinical applications, where disposable components are usually preferred. Coatings for both multiple and single-use applications require a certain stability, be it for multiple measurements or simply for storage. This issue is not yet sufficiently investigated in the development of new coatings [3,5,16,53,61].

### 2.3. Array Designs

**Figure 3:**
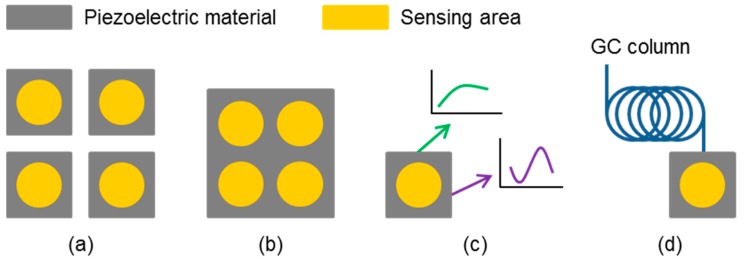
Acoustic array designs: (**a**) Modular multi-sensor array; (**b**) monolithic multi-sensor array; (**c**) virtual sensor array based on different signal responses obtained from a single sensor; and (**d**) virtual sensor array based on the combination of sample separation by gas chromatography (GC) and subsequent peak detection by a single sensor.

The design of a multi-sensor array typically includes spatial proximity of the sensor devices, particularly if the sample volume is limited. Since acoustic sensor devices are operated at high frequencies ranging from MHz to GHz, multiplexing techniques are recommended to avoid interference and crosstalk resulting from this closeness as they would affect the signal responses [75,76]. Figure 3 depicts basic array designs for multi-analyte detection realized with BAW and SAW sensors. The setups can mainly be divided into modular, monolithic, and virtual sensor arrays. 

Figure 3a shows the most common form of an array, namely the combination of several sensor devices into a modular multi-sensor array. In the modular setup, the sensors can be coated and assembled individually according to application. Defective parts can easily be replaced. Configurations include the combination of single-sensor devices in one measurement cell, the combination of measurement cells containing single sensors, and the combination of disposable sensor chips, i.e., single sensor devices with polymer housings [77]. When working with large-volume gas samples, several single-sensor devices can be exposed to the gases in a comparatively large measurement chamber as has been realized for QCM, FBAR, and SAW sensors [21,78,79]. With reduced sample volumes and particularly with liquid samples, however, the volumes required for uniform sampling of the sensors would be too high in the chamber setup. Therefore, providing sensor devices with a measurement cell or polymer housing with flow channel leading the sample flow near the sensors would be more suitable for samples with limited volume. Gaseous samples would allow measurement cells containing several sensor devices [80], but this is not suitable for liquid samples because of uncontrollable leakage in between the devices. Measurement cells with one or more sensor device(s) are typically designed for simple sensor replacement, i.e., the flow cell itself is made for repeated use. In contrast to that, sensors with polymer housings are rather intended for use as disposable components as they combine low-cost sensor devices with generally economic packaging materials and procedures. Furthermore, such devices are easier to miniaturize than flow cells with replaceable components. Both flow cells and sensor chips must consider array compatibility in their design to reduce dead volumes and, hence, sample consumption. Otherwise, the connection of single components to arrays will increase the sample volume disproportionally, which is an issue particularly when working with liquid samples, such as body fluids for biomedical applications [3,77]. 

Close sensor-to-sensor connection is given already in monolithic multi-sensor arrays (Figure 3b), which have been realized for QCM, FBAR, and SAW sensors [52,81,82,83]. Since in principle the sensing elements in monolithic arrays can be brought together more closely than in modular arrays, this approach has a high potential for miniaturization. On the other hand, if the sensing areas are to be coated and connected individually, spatially resolved surface functionalization is essential, making the coating procedures often more complex than required for modular components [52]. Furthermore, if one sensing structure is defect, the complete array may have to be discarded. 

Despite the common use of multi-sensor arrays, they still have some unresolved limitations. First of all, the set of coating materials in an array must individually be adapted for each application. In addition, sensor drifts interfering with the sensor signals may be different for each sensor and each coating [84]. Therefore, the use of less sensors per array would be advantageous, provided that the required information content is still available. This is fulfilled by virtual sensor arrays (Figure 3c) realizing a different approach for multi-analyte detection. Instead of increasing the degree of parallelization, they use multiple signal responses extracted from one sensor device. This includes, for instance, the evaluation of a signal response curve regarding both signal shift and response time. Newly developed signal processing and evaluation methods even allow detection and quantification of complex mixtures from one signal response curve [71,85,86]. Furthermore, dual or multiple signal transductions have been exploited, e.g., by evaluating both phase and attenuation shifts of a SAW sensor [87], by determining SAW sensor frequency shifts at different temperatures [88], or by measuring the frequency and dissipation shifts of a QCM sensor at multiple harmonics [89]. When conductive films are used as sensor coatings, changes of both the acoustic wave and the electrical properties of the coating can be evaluated [90,91,92]. As both multi-sensor arrays and virtual sensor arrays turned out to be well suited for multi-analyte detection, both were combined to virtual multi-sensor arrays to further enhance the performance [93,94].

Another possibility for multi-analyte detection and quantification with a single acoustic sensor is the combination of the sensor with a GC column as shown in Figure 3d. This e-nose is also known as virtual sensor array [95] and has been realized with both QCM and SAW sensors (see Section 3.1.5). The sample components are separated by the GC column and can be identified via the retention time. Subsequent quantification is done by mass adsorption on the acoustic sensor. A sensor coating may be applied but is not required for identification. Therefore, uncoated sensors can also be used, avoiding any interference from potentially instable layers. GC-SAW instruments with uncoated SAW devices have been commercialized (see Table 2).

### 2.4. Sampling

**Figure 4:**
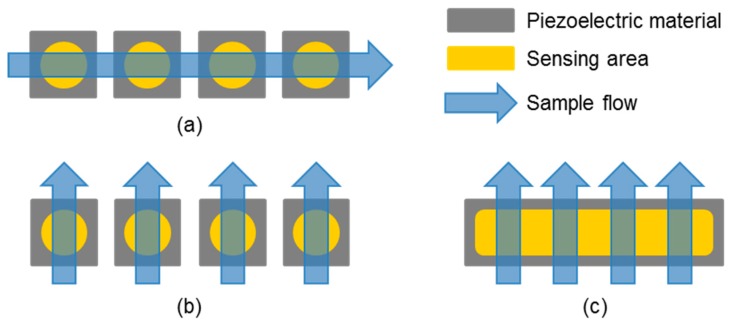
Sampling schemes: (**a**) Serial application of one sample on several sensing devices; (**b**) parallel application of one or more samples on several sensing devices; and (**c**) serial application of several samples on one sensing device (e.g., µF-on-SAW [101]).

As mentioned in the section before, gas sensors can be operated in comparatively large measurement chambers, allowing the gas to flow over or around the sensors. It has to be ensured that the volume is large enough for the gaseous medium to be equally distributed, which can easily be obtained, e.g., by a fan [78]. Such chambers are less common for liquid samples where the sample volume is typically limited. A beaker-like setup with 3 × 3 QCM sensors at the wall and a stirrer to distribute the liquid was introduced for immunoassays, but measurements were not included in this study [96]. Gas sample volumes, however, can also be limited, for instance, when only the headspace over a liquid or a solid sample is available or when sample enrichment is required because of low analyte concentrations. Sample enrichment can be obtained, for instance, by solid phase microextraction (SPME) using an adsorbent-coated fiber or by utilizing a so-called trap. A trap describes a pre-concentration unit consisting of a tube (e.g., glass or polytetrafluoroethylene) filled with sorption material. Subsequent heating of the fiber or the trap allows the volatile sample components to desorb and to be led to the sensors [97,98,99,100]. Samples with limited volume require a more directed flow leading the sample to the respective sensors. This can be realized with reduced chamber volumes down to flow channels, as typically provided by flow cell setups. Examples of sampling by flow channels are depicted in Figure 4. 

Serial sample application (Figure 4a) is the most commonly used sampling scheme. Large arrays are sometimes split into two rows of sensors, allowing more compact setups with either one channel in between or a split channel for each row of sensors [22,38]. Even though gases spread faster than liquids, designs must consider and avoid potential turbulences to guarantee stable and consistent operation of the array [102,103]. Laminar flow conditions are particularly essential for liquid samples. For instance, a circular flow chamber on a monolithic QCM with 2 × 2 sensing areas showed a highly turbulent flow leading to inconsistent sensor responses. A fluidic channel addressing the sensing areas subsequently was required to obtain reproducible results [104,105]. 

Serial sampling has led to negligible delays in the signal responses of gas sensors. As shown for SAW sensors, signal responses can further be optimized when the SAW devices are capacitively connected to the electronics via contact pads beside the fluidic channel instead of using sockets, such as the TO39 housing, because the glue of the housings may serve as an additional absorbance layer interfering with the sensor results when gases are released. The miniaturization of the flow cell showed improved flow profiles for both gas and liquid applications [80]. Further miniaturization was obtained by manufacturing polymer housings for SAW biosensors in a way that eight of the resulting SAW biosensor chips were combined with a microfluidic chip to an eight-channel array. A tailor-made microfluidic setup restricted the media contact of multi-use active components, such as pumps and valves, to a passive, intermediary liquid. Only the disposable components, i.e., biosensor and microfluidic chips, were in contact with the samples, which made the setup highly suitable for biomedical applications. However, despite the improved flow channel reducing the dead volume to the first biosensor chip to a minimum, serial sampling of the eight biosensor chips led to a considerable delay between first and last sensor [77,106]. In addition to the delay between the signal responses, another disadvantage of serial sampling is the potential risk of sample depletion by cross-reactive binding on the first sensors, leading to reduced signal responses of the later sensors. This could be circumvented by parallel sampling (Figure 4b), but design and fabrication of the fluidic system would be more complex, as signal interferences resulting from changes in the flow conditions must be avoided. A parallel setup for SAW biosensor chips has been set up but not tested yet [107]. As far as miniaturization is concerned, FBARs again have an advantage here, as they can be manufactured via CMOS techniques, and microchannels can be integrated in the same process [108]. 

A—serial-parallel fluidic combination leading to another form of an array for multi-analyte detection is depicted in Figure 4c. This sampling scheme was realized using two-port delay-line-SAW devices; therefore, this concept has been called “µF-on-SAW”, i.e., “microfluidics-on-SAW”. The setup consists of several flow channels crossing the path of the delay lines. Subsequent sampling of the channels allows serial recording of signal responses depending on the surface functionalization [101]. Exemplary applications using this technique are summarized in Section 3.3.3.

### 2.5. Commercially Available Acoustic Sensor Array Instruments and E-Noses

Table 1 summarizes commercially available acoustic sensor arrays utilizing QCMs. They are mainly designed for liquid applications, but they can also be used for gaseous samples if an appropriate sampling unit is provided as shown, for instance, for the QSense system from Biolin Scientific [25]. All arrays use the resonance frequency as signal response; many include the dissipation values. The fundamental frequencies of the QCM sensors are in the typical range of up to 50 MHz. AWsensors stands out here, as their model AWS A20+ RP additionally offers QCM sensors with high fundamental frequencies of 50 MHz, 100 MHz, and 150 MHz. Furthermore, this instrument can be equipped with SAW sensors supporting Love waves at 120 MHz. Apart from the latter, no SAW sensor array instruments for liquid applications are currently commercially available (regarding e-noses, see Table 2). The S-sens K5 was an instrument based on a monolithic array with five SAW delay lines supporting Love waves. It had been introduced by the Center of Advanced European Studies and Research (caesar) in Bonn, Germany [83] and was commercially available until recently as “Seismos” from Nanotemper (Germany) [7]. However, according to the current status, the instrument is no longer on the market and only the Seismos SAW sensor chips (i.e., the monolithic arrays) are still available [109].

The QCM sensor array instruments in Table 1 are mainly designed for use in research laboratories. The arrays are typically based on modular setups, i.e., the QCM crystals are located in individual flow cells. For multi-sensor measurements, several flow cells have to be connected serially or in parallel with an appropriate fluidic system. In contrast to that, Initium offers two systems, Affinix Q8 and Affinix Qµ, with so-called “cup-typed” sensor cells resembling microplate wells with the QCM sensor at the bottom. The liquid samples can be pipetted in those cells, e.g., with an eight-channel pipette. Nihon Dempa Kogyo offers the NAPiCOS series with monolithic twin-QCMs, i.e., a quartz crystal with a sensing and a reference electrode, where the latter can be coated with a reference layer, such as a blocking chemical. 

Commercially available e-nose instruments based on acoustic sensors are listed in Table 2. They range from instruments for research laboratories (e.g., SAGAS (Surface Acoustic Wave Aroma and Gas Analysis System)) to devices ready for the end user (zNose and HAZMATCAD (Hazardous Material Chemical Agent Detector). While QCM sensors predominate in acoustic sensor arrays for liquid applications (see Table 1), both QCM- and SAW-based instruments are available as e-noses. Until recently, there was also LibraNose, a QCM-based e-nose developed at the University of Rome Tor Vergata and distributed by Technobiochip, Italy [10,110]. However, the company no longer seems to exist and, therefore, the instrument is no longer available. 

Interestingly, commercial e-nose instruments are not necessarily based on multi-sensor arrays. The e-noses from Nihon Dempa Kogyo (Twin-CQCM and Twin-TQCM) each provide a monolithic quartz crystal with a sensing and a reference electrode, similar to the setup for liquid samples (see Table 1). However, in the e-nose setup, the reference electrode is shielded from the environment and only the sensing electrode is accessible for the sample. Both the FDM (Fuel Dilution Meter) 6000 from Spectro Scientific and the zNose series from Electronic Sensor Technology use single SAW sensors for their e-noses (see Table 2), utilizing the potentially higher operation frequency and, hence, sensitivity of the SAW sensors compared to the QCMs. The FDM 6000 contains only one polymer-coated SAW device intended for fuel detection in the headspace of engine oils. As this describes a limited application, it may be assumed that no other contaminants are present and, therefore, that one sensor is enough to obtain accurate results. The zNose series includes both benchtop and portable devices. They have in common that GC is combined with SAW sensor technology, i.e., the components provided by the gaseous sample are separated by GC and subsequently quantified by the SAW sensor. Uncoated SAW devices are used, and the identification of the components is made possible by databases for the GC peaks. 

In this section, acoustic sensor arrays used for multi-analyte detection are described for applications in gaseous and in liquid media. Both multi-sensor and virtual sensor arrays are shown. The sensors used in the following were based on common piezoelectric materials, i.e., QCM sensors on AT-cut quartz; FBARs on ZnO and AlN; and SAW sensors on ST-cut quartz, LiNbO_3_, or LiTaO_3_. 

## 3. Multi-Analyte Detection with Acoustic Sensor Arrays

### 3.1. Acoustic Gas Sensor Arrays: E-Noses

The following overview focuses on the e-nose developments of the past decade. The sensor array applications are grouped according to the sensor coatings and within the groups mainly according to decreasing number of sensors in the array. 

#### 3.1.1. QCM Multi-Sensor Arrays 

LibraNose, an e-nose system which was commercially available until recently (see Section 2.4), provided an e-nose based on eight QCMs which were coated either with polypyrrole polymers modified by aldehydes or with metalloporphyrins. A study on recognizing incipient wood decay caused by fungal infestation compared both sets of coatings. Volatile profiles of ten healthy wood types and wood decayed by nine different fungi were compared. Both coating sets allowed clear discrimination between healthy and decayed wood, with the polypyrrole coatings showing the best results [111]. Furthermore, polypyrrole and metalloporphyrin coatings were applied for the detection of meat spoilage, which would be indicated by the volatile profile of the microbial population. Both sets allowed the biochemical signatures to be evaluated in a way that both the degree of freshness/spoilage and the microbial load of the respective meat could be predicted to a good extent [112,113]. 

An array of eight QCM sensors coated with seven metalloporphyrins and the free base of a functionalized porphyrin was used to identify twelve microorganisms, including eleven bacteria and one fungus. The assignment of the results to blank culture media and microorganisms was unambiguous. Furthermore, Gram-positive bacteria and Gram-negative bacteria could be distinguished [114]. If the metalloporphyrins were grafted on ZnO nanorods using different procedures, an increase in sensitivity and selectivity was obtained compared to the porphyrin alone. It could be shown that three of such coated QCM sensors, including ZnO-free porphyrin, were sufficient to separate culture medium from cells and two different cell lines. Using four of those sensors, including ZnO-free porphyrin and porphyrin-free ZnO, allowed the separation between four classes of VOCs (alcohols, amines, aliphatic, and aromatic hydrocarbons) from which six compounds were applied [115]. An array of six metalloporphyrins varying in the metal ion only was successfully used to identify six VOCs from six different classes, including an aliphatic and an aromatic hydrocarbon, an alcohol, a carboxylic acid, an amine, and an organosulfur compound [116]. An array of eight QCM sensors coated with metalloporphyrins for the detection of three aliphatic compounds was used to define calibration procedures as preparation for disease studies using breath samples [117]. In the following, similar arrays were used for early diagnosis of lung cancer and for tuberculosis diagnosis. The exhaled breath of lung cancer patients and from patients with pulmonary tuberculosis could be distinguished from the corresponding samples of healthy controls with sufficient selectivity [40,118]. 

An array of eleven QCM sensors coated with nine metalloporphyrins and two corroles was used to detect malaria in the total mouse volatilome. Infected mice were correctly identified if the parasite infestation was not too low [119]. An array of eight QCM sensors, where three were coated with metalloporphyrins and five were coated with polymers, was used to determine biogenic volatiles released by soil to measure the microbial activity. Distinct soil volatile profiles could be determined, but the clear differentiation between sterilized soil inoculated with microorganisms and non-inoculated control soil proved to be difficult, probably because of abiotic processes contributing to the soil volatiles [120]. 

An array of nine QCM sensors coated with different phthalocyanines, including complexes and derivatives, was used to detect wastewater odors. The sensors showed reversible signal responses to odorous substances like organic amines and organosulfur compounds. Characteristic profiles for sewage samples were obtained; however, they could not be clearly assigned to the stage of the wastewater treatment plant where the samples had been collected [44]. An array of eight QCMs coated with fluorinated and non-fluorinated phthalocyanine complexes was used for the detection of twelve VOCs, including aliphatic and aromatic compounds, of which some were chlorinated. The fluorinated alkyloxy substituents were particularly suitable for the selective detection of the polar VOCs while the humidity influence remained moderate [121]. Hybride and nanocomposite coatings were applied on a monolithic three-channel QCM array. A metallophthalocyanine with silica hybrid film and a metal oxide with MWCNT nanocomposite were applied on two of the three sensing areas while the third was left blank as reference. The array was successfully used for the selective detection of acetone and nitric oxide (including mixtures) as volatile biomarkers for potential application in early asthma and diabetes diagnosis [122]. 

GUMBOS compounds based on metallophthalocyanine tetrasulfonate were applied on the QCM sensors of a four-channel array (Biolin Scientific; see Table 1). The array allowed sufficient discrimination between ten different VOCs into the corresponding functional group classes (alcohols, aromatic and aliphatic hydrocarbons, and chlorinated hydrocarbons), which was promising for applications in food quality control [25]. An array of eight QCM sensors coated with different ionic liquids was applied to detect three explosives including methane and two aromatic nitro compounds. Both single components and binary mixtures could reasonably be discriminated [123]. Furthermore, seven ionic liquids were applied on the sensor surfaces of an array to analyze 31 VOCs from nine different classes, including alcohols, phenols, acids, esters, aldehydes, ketones, amines, hydrocarbons, and terpenes. The chemical classes could clearly be discriminated. As the chosen VOCs represented a wide variety of food flavors, the array should allow the estimation of food quality and origin, which was successfully demonstrated by the differentiation between the aroma profiles of two botanical varieties of cinnamon [42]. 

An array of five QCM sensors was coated with three ionic liquids and one GC stationary phase while one sensor was left uncoated as reference. This array was successfully used to identify four VOCs from four classes, i.e., an alcohol, a ketone, a chlorinated hydrocarbon, and an aromatic hydrocarbon [124]. Similar to that, a monolithic QCM sensor array was described using two ionic liquids and a conductive polymer. With this array water and three aliphatic VOCs, an alkane, an alcohol, and a chlorinated hydrocarbon could be discriminated [125]. 

Six QCMs were coated with MWCNTs subjected to different treatments. This array was used to detect twelve aliphatic alcohols and eight aromatic hydrocarbons. However, the frequency shifts were not sufficient to differentiate within the compound families. Higher selectivities were obtained when the sorption times were also adapted [27]. Furthermore, MWCNTs were part of an array with four diversely coated QCM sensors. The sensors were coated in a way that highly selective affinities were obtained to hydrogen sulfide (copper oxide coating), ammonia (polyaniline coating), and dimethylamine (MWCNTs and graphene coating), i.e., compounds representing volatiles emanating from eggs. In the end, batches of fresh eggs and of eggs stored one, two, or three weeks could effectively be differentiated from each other [126]. Another array using diverse coatings was based on three QCM sensors and coatings of graphene oxide, graphene oxide functionalized with β-cyclodextrin, and a composite of gold nanoparticles (AuNPs) with N-functionalized pyrrole. Three inorganic toxic gases could reasonably be identified not only as single components but also in tertiary mixtures [78]. 

Polymers, lipids, macrocyclic compounds, and biochemical reagents were considered as coatings for arrays of eight QCM sensors. Such assorted arrays were used to detect foodstuff adulterations by synthetic flavoring agents and release of aliphatic and aromatic compounds from polymers used in household products [127,128]. An array of eight QCM sensors was coated with polymers, metal chlorides, composites, and an antibiotic to determine volatile profiles of Chinese liquor samples. It was possible to classify ten liquors with good accuracy and to reliably differentiate between twelve liquors according to flavor type [129,130]. A QCM sensor array consisting of four sensors coated with polyethylene glycols of different molar mass and four sensors coated with glucose derivatives, including D-glucose, maltose, maltodextrin, and β-cyclodextrin, was used to monitor black tea fermentation. The optimum fermentation times of twelve black tea cultivars determined by the QCM sensor array were in good agreement with the results obtained with the reference method based on ultraviolet-visible (UV-VIS) spectrophotometry [131]. 

Six polycyclic aromatic hydrocarbons were used as coatings within an array of six QCM sensors. Nine VOCs of different polarity, including alkanes, ether, alcohols, and aromatic hydrocarbons, were detected with good sensitivity and selectivity while the frequency shift remained relatively stable over a wide range of humidity [132]. Eight anthocyanins were used as array coatings for breath analysis. The array was calibrated to 15 alkanes, eight alcohols, twelve aldehydes and ketones, five chlorinated compounds, and twelve aromatics and terpenes. The study showed that a thermal desorption process of the VOCs adsorbed on the collection cartridge may serve as a pre-separation step. In a first test, chronic obstructive pulmonary disease (COPD) patients were perfectly discriminated from control individuals [133]. An array of nine QCM sensors was coated with different composites including permanent marker liquids. This setup yielded satisfactory results for the selective detection of individual gas concentrations in binary mixtures of three VOCs, i.e., a ketone, an alcohol, and a chlorinated aliphatic compound [134].

MIPs for highly selective terpene detection were prepared for two monolithic quartz arrays carrying three sensing areas each, i.e., six sensing channels were available. Two sets of six MIPs were prepared for time-resolved monitoring of terpene emanation from either fresh basil and peppermint or fresh and dried basil, rosemary, and sage. The results of terpene progression were similar to results obtained with GC-MS [135,136]. A binary array coated with MIPs selective to benzene and isopropyl methyl ketone allowed the satisfactory quantification of binary mixtures of the corresponding analytes [137]. Furthermore, molecularly imprinted materials were used for the detection of VOCs representing body odors. A QCM sensor array using three MIPs was developed for the selective detection of organic acids, while QCM sensor arrays using three MIPs and one reference (non-imprinted polymer) were used for the selective detection of aldehydes. When real body odor samples were tested, the presence of all target analytes detected by the QCM sensor arrays was confirmed by GC-MS measurements [138,139,140]. It could be shown by the selective detection of aldehydes with an array that molecularly imprinted sol-gels offer a promising alternative to MIPs [141]. Furthermore, utilizing a similar array with coatings made of MIP nanobeads for organic acid detection resulted in enhanced sensitivity and selectivity because of the nanobead structures [142]. 

AuNPs functionalized with hairpin DNA loops were used as sensing elements for VOC detection. An array with eight QCM sensors could discriminate four molecular classes, as shown with eight VOCs, including alcohols, esters, aldehydes, and ketones, and separate VOC molecules by molecular weight. Furthermore, seven VOCs representing the aroma of carrots could well be discriminated. The volatile profile in real carrot samples detected with the sensor array was similar to the profile obtained with GC-MS. Changes in the aroma profiles of the samples resulting from different storage times and temperatures could be detected by the array [29,143]. 

An array providing 24 peptide-coated QCM sensors was used for breath analysis to identify bacterial infections in patients with assisted breathing. Though the underlying chemical species responsible for the sensors’ responses had not been identified before, six different bacterial pathogens could be identified in the breath samples with good accuracy. The results were validated with cultures from sputum samples of the same patients [144]. Peptides for chemical sensing were later introduced on an array with up to eight QCM sensors. It was advantageous to introduce the peptides as peptide-functionalized AuNPs instead of immobilizing monolayers of peptides as the use of the AuNPs increased the sensitivity by two orders of magnitude. Aside from VOC discrimination and detecting food aromas in different solvents, arrays with peptide-functionalized AuNPs differentiated satisfactorily between extra virgin and virgin olive oils [30,145,146]. Using ZnO nanoparticles instead of AuNPs led to similar results regarding the discrimination ability between five alcohols and three esters. Furthermore, good results were obtained for distinguishing between the aroma profiles of water and fruit juices, where the latter is influenced by fruit and sugar content [147]. Both an array with eight metalloporphyrin-coated QCM sensors and an array with eight AuNP-peptide-coated QCM sensors were able to discriminate standard chocolate samples from artificially degraded chocolate samples with good accuracy, albeit the peptide-coated sensors showed a better prediction performance [148].

#### 3.1.2. FBAR Multi-Sensor Arrays 

The potential of an FBAR array with six polymer coatings for indoor air-quality monitoring was demonstrated by the ability of this array to distinguish between four VOCs representing four classes, i.e., an alcohol, an ester, and a ketone, and an aromatic hydrocarbon [149]. An FBAR array with two polymer coatings was successfully applied for quantitative detection of three alkanes and one ketone after GC. Furthermore, binary mixtures of one of the alkanes and the ketone (i.e., pentane and acetone), which could not be separated by the GC column, could be differentiated by the array [46].

An array of nine FBARs coated with silane self-assembled monolayers, partially enhanced with polyethylene glycols, was successfully used for the selective detection of one ketone and four alcohols and for interaction studies of the VOCs with the different chemical groups on the surface [21]. Similarly, an array of four FBARs, each one coated with another type of supramolecular monolayer, was used for the selective detection of six aliphatic compounds from four classes, i.e., two hydrocarbons, one chlorinated hydrocarbon, two alcohols, and one ketone. Furthermore, kinetic and thermodynamic constants were calculated out of the response curves to quantify the interactions between the respective gas molecules and supramolecular monolayers [37]. 

#### 3.1.3. SAW Multi-Sensor Arrays 

SAW multi-sensor arrays in the past decade were mostly based on Rayleigh waves on ST-cut quartz or 128° YX-LiNbO_3_, but STW devices on 36° Y-cut quartz devices have also been reported. 

An STW quartz two-port resonator array (SAGAS-type instrument; see Table 2) using eight polymer-coated sensors detected the changes in the aroma profile of coffee powder caused by ageing up to 14 days [43]. A similar array with polymer-coated STW resonators was used to test the long-term stability of the polymer layers. It was shown that the ability of the polymer layers to distinguish between three VOCs of different classes, i.e., a chlorinated aliphatic compound, an aliphatic hydrocarbon, and an aromatic hydrocarbon, was maintained for at least three years. Furthermore, the ability to distinguish between similar VOCs from the same class, i.e., between two aliphatic hydrocarbons or two aromatic hydrocarbons, lasted for at least one year [38,150]. 

Other stable coatings can be provided by diamond nanoparticles as shown by an array of eight SAW quartz two-port resonators (SAGAS instrument; see Table 2). Post-treatment of these surfaces by oxidation or reduction allowed the selective detection of both inorganic and organic vapors, i.e., ammonia, an alcohol, a nitroaromatic, and an organophosphonic compound [28]. Furthermore, the use of these surfaces as intermediate layers offers functional groups for covalent coupling. The covalent immobilization of six major mouse urinary proteins allowed the selective detection of two nitroaromatic compounds which may occur in explosives [31].

An array of six SAW quartz resonators coated with phthalocyanines was used to selectively detect six aliphatic compounds with particular emphasis on the differentiation between acetone and trichloroethylene [151]. Another array of six SAW quartz two-port resonators using five different polymer coatings and one uncoated reference allowed the discrimination between three CWA simulants, an ester, and water [152].

An array with five SAW quartz dual two-port resonators containing four polymer coatings and one uncoated reference was used on 14 different volatile blends made of an alcohol and an ester having a similar molar mass. The ratiometric information could successfully be collected and evaluated, paving the way for the development of an info-chemical communication system [153]. Another five-channel two-port SAW resonator array was provided with four sensors carrying coatings from different classes (triethanolamine, polyepichlorohydrin, fluoroalcoholpolysiloxane, and L-glutamic acid hydrochloride) and an uncoated reference sensor. This array was successfully used for the highly selective detection of two harmful inorganic gases and two CWA simulants by wireless communication technology within a communication distance of 300 m. The sensor array system was also equipped with a GPS (global positioning system) module to determine the location of the measurement [154]. 

Polymers were used to coat three out of five SAW quartz two-port resonators, while two resonators were left uncoated. The resulting array was successfully applied to selectively detect three CWA simulants and the CWA sarin [155]. The same array setup but with three types of odorant-binding proteins instead of polymers was utilized to differentiate between a terpenoid and a mushroom alcohol, which could be used to assess indoor air-quality or food contamination [33].

An array of four SAW quartz one-port resonators coated with metal oxides was successfully used to detect and discriminate four CWA simulants even in the presence of four interfering substances (three fuels and acetone). Furthermore, binary mixtures of one CWA simulant and an alcohol could clearly be recognized [39,156]. An array with four polymer-coated SAW resonators was introduced for the selective detection of solvent vapors in breath and ambient air. This was further developed into a GC-detection array. Daily calibration with a mixture of C6 to C22 n-alkanes allowed the highly selective detection of volatile biomarkers representing active pulmonary tuberculosis in picomolar concentrations [157,158]. A set consisting of five polymer-based adsorbents and one cryptand was evaluated with an array of two SAW quartz one-port resonators for application in polymer plants. The best pair of polymers enabled clear discrimination between an inorganic and an organic volatile, i.e., carbon disulfide and methanol [159].

SAW quartz delay line sensor arrays were developed with and without wave-guiding layers. An array of seven sensors, six coated with rubber-like and amorphous polymers and one left uncoated as reference, was successfully used to discriminate between six CWA simulants and toluene as reference with good detection limits in the sub-ppm range [160]. The latter was improved by introducing novolac or quartz as guiding layers to obtain Love waves with higher sensitivity [161,162]. A similar quartz Love wave sensor array but with polymer nanofibers, including polymer nanofibers with metal content, allowed excellent discrimination of four CWA simulants [163]. The array setup was also used with ZnO as both guiding and sensitive layers. Further coatings were performed with metal oxide nanoparticle layers which were partially enriched with different metals. This array allowed the differentiation between ammonia and two aromatic hydrocarbons [164]. 

An array of five quartz delay line sensors with similar nanocomposite coatings was used for selective detection of three CWAs. The coatings were metal oxides and nitrides embedded in polymer in addition to the plain polymer as reference sensor coating. The polymer coatings containing nanoparticles yielded higher signals than the pure polymer [165]. Another array of five quartz delay line sensors was coated with two polymers containing two different percentages of MWCNTs while one sensor was left uncoated as reference. High, distinguishable responses were obtained for an aliphatic and an aromatic hydrocarbon, whereas the array yielded no responses to inorganic gases [166]. 

SAW sensor arrays based on LiNbO_3_ also included both delay line and resonator devices. In the following examples, all sensors were coated with polymers except one uncoated reference sensor per array. SAW LiNbO_3_ delay line sensors were combined into 2 × 2 arrays to selectively detect five volatiles, including two alcohols, two amines, and acetone. A further development allowed the wireless readout of this setup, e.g., for wireless sensor network applications [47,167]. A low noise CMOS readout circuit was developed for an array consisting of five SAW LiNbO_3_ resonators for differentiation between two aliphatic alcohols [79].

#### 3.1.4. Acoustic Virtual Sensor Arrays

A polymer-coated QCM sensor was used to detect four aliphatic oxygen-containing compounds by utilizing both frequency shift and response time. The polyethylene glycol (PEG) coating allowed discrimination between the alcohol, the ester, and the group of two ketones. However, both of the ketones yielded similar signals and, therefore, could not be distinguished from each other. [85].

Another virtual QCM sensor array exploited the frequency shifts at several harmonics. Ionic liquids were tested as coatings for this array. The appropriate coating and coating thickness allowed both interclass and intraclass classification of 18 VOCs of four classes (aromatic and aliphatic hydrocarbons, aliphatic chlorinated hydrocarbons, alcohols, and nitriles) with an accuracy of almost 100% [84]. The same measurement method was performed with a QCM sensor coated with a binary blend made of an ionic liquid and a polymer, enabling the discrimination of eight closely related alcohols. Additional evaluation of the respective dissipation values allowed the approximation of the molecular weights from the quotient frequency shift by dissipation shift [89]. This virtual sensor array consisting of one QCM was enhanced to a virtual multi-sensor array, i.e., four QCM sensors were coated with different ionic liquids, and frequency shifts were evaluated at multiple harmonics for each QCM. This setup was successfully used to discriminate four different petroleum-based fuels and three gasoline grades. Furthermore, several grades of gasoline contamination by organic solvents (1% to 40% alcohol or aromatic hydrocarbon) could be estimated, including the nature of the contaminant [94]. A similar setup was applied to identify five citrus-scented odors. It could be shown that virtual sensor arrays and multi-sensor arrays yielded comparable results. The identification accuracy was below 100% for both arrays but could be increased to 100% by combination of the arrays to virtual multi-sensor arrays. This confirms that this new approach of virtual multi-sensor arrays is highly promising, particularly if complex mixtures are to be identified [93]. 

Virtual FBAR arrays using dual signal transduction were recently introduced. The FBARs were coated with conductive polymer films, and combinations of frequency with resistance or impedance readouts were evaluated, partly with additional modulation of the temperature. These setups allowed the differentiation between five aliphatic compounds or one aromatic and five aliphatic compounds [91,92]. A similar approach was performed when a one-port SAW resonator made of 128° YX-LiNbO_3_ was coated with a conductive material. Parallel to the frequency change, variations of the conductive polymer’s resistance were measured, the latter through the two terminals of the IDT. Three aliphatic compounds from three different classes were distinguished by this setup [90]. Furthermore, the frequencies of a polymer-coated SAW quartz one-port resonator were recorded at different temperatures in the range of −20 to 70 °C. Characteristic signal patterns were obtained for harmful vapors of different VOC classes, i.e., an alcohol, an aromatic compound, an organophosphonate, and diesel fuel [88]. 

#### 3.1.5. Acoustic QCM and SAW Single Sensors Combined with GC or SPME

As shown in Figure 3, a single sensor combined with GC may also act as an e-nose for multi-analyte detection. Over the past decade, such e-noses have been predominated by GC-SAW setups, probably because of their commercial availability (zNose series; see Table 2). 

GC-QCM was used to classify 16 odors, including organic solvents, fuels, insecticides, and perfumes. Subsequent training of a neural network to recognize these odors resulted in an identification rate of 85% [168]. A QCM sensor was coated with 1,10-decanedithiol and connected to a SPME fiber to follow the degradation of butter. SPME-GC-MS measurements revealed 13 major volatile compounds during the degradation process which were all detected by the SPME-QCM system, albeit with different sensitivities. However, both setups detected 2-heptanone, which is a good marker for butter oxidation for the first three weeks [169]. Another QCM sensor was subsequently coated with a polymer and a carbonaceous nanomaterial, where the latter increased the sensitivity of the QCM up to three orders of magnitude compared to the uncoated device. GC-QCM measurements could be performed at temperatures above 100 °C and allowed the determination of eight illegal drugs from sample collection cotton swabs with the lowest amounts of detection ranging from 0.04-3 µg, according to the substance [170].

Applications of GC-SAW instruments from the zNose series include the following:

- Breast cancer risk prediction based on volatile biomarkers in breath samples. The zNose results were similar to results obtained with GC-MS [171].
- Quality control of medicinal plants based on differences in the herbal aroma components. Several plants of the Lavandula species, including lavenders and lavandins, could clearly be identified and distinguished from each other [172], as could different plant parts (leaves and arial and underground stems) of Houttuynia cordata Thunb [173].
- Characterization of fruit ripening and aroma quality based on volatile profiles. Evaluation of mango maturation was successfully combined with rot prediction to estimate the shelf life of the fruit [174]. Melons harvested at different stages of ripeness (early and full) could be distinguished from each other [175]. Several blueberry cultivars were classified according to their genotypes or their degree of ripeness [176].
- Classification and quality control of processed foods based on volatile profiles. Fatty acids determine the aroma profile of fats, lards, and oils. Pure animal body fats and lards with varying fat contents could be distinguished from each other and from adulterated samples. Adulteration of lard with chicken fat could be detected down to an impurity level of 1% [177]. Contaminations of virgin coconut oil with palm kernel oil were detected down to an impurity level of 1% [178]. Turkish extra virgin olive oil samples could be classified according to cultivar, geographical origin, and harvest year [179]. Cabernet Franc and Merlot wines were identified with regard to different canopy sides. Furthermore, it could be detected whether the grapes were treated with ethanol at the beginning of ripening. These differences were not necessarily detectable in sensory tests, showing that the e-nose recognizes both aroma and non-aroma volatiles [180,181].

### 3.2. Acoustic Liquid Sensor Arrays: E-Tongues

E-tongue development in the past decade has mainly included QCM multi-sensor arrays and SAW virtual arrays.

#### 3.2.1. QCM Multi-Sensor Arrays 

An array of four QCM sensors coated with different phthalocyanines was successfully applied to discriminate between four pesticides from four common classes (organophosphate, carbamate, pyrethroid ester, and azole) in water with limit of detection (LOD) values below 0.09 mg/L (0.09 ppm) [49]. An array with three similarly coated QCM sensors was used to detect and distinguish three organic solvents (one aromatic and two chlorinated aliphatic hydrocarbons) in water. Both pure substances as well as binary mixtures were applied in concentrations ranging from 14 to 990 ppm and could be identified with average prediction errors below 5% [182]. Eight QCM sensors were coated with polymethyl methacrylate (PMMA)-plasticizer films using varying plasticizer contents. The sensors were operated in two arrays at four sensors to detect and sufficiently discriminate five aromatic hydrocarbons in water at concentrations up to 100 ppm, representing water contamination by petroleum hydrocarbons [50].

#### 3.2.2. SAW Virtual Sensor Arrays 

SAW sensors in the following were based on 36° YX-LiTaO_3_. 

Uncoated SH-SAW two-port resonators were used as single sensor devices to investigate liquid samples representing six tastes, i.e., salty, sweet, sour, bitter, umami, and metallic. Phase and attenuation values were recorded and plotted against each other, allowing the classification of the 0.1 M taste samples and the recognition of binary taste mixtures [87].

A polymer-coated SH-SAW delay-line device was used to detect several ppm concentrations of two organophosphate pesticides in water. Using both frequency shifts after the equilibrium is reached and the time constants associated with the adsorption process allowed the differentiation between the two compounds [71]. SH-SAW dual delay-line devices were used as single-sensor devices as one line served as a sensing line and the other served as a reference line. Polymer coating of the sensing line and the exploitation of both equilibrium frequency shifts and response times enabled qualitative and quantitative determination of aromatic compounds and compound mixtures in water at sub-ppm concentrations. As the signals of the structural isomers ethylbenzene and *o*-, *m*-, and *p*-xylenes were similar, only a combined concentration for those components in mixtures could be given. Aliphatic components, however, did not interfere with the signal responses. In mixtures, the concentrations obtained with the sensor were comparable to those obtained with GC-PID (GC with photoionization detector), with the average difference being ±6.3% [72,86].

### 3.3. Acoustic Biosensor and Chemosensor Arrays 

Biosensor and chemosensor applications require functionalization with specific recognition layers. Consequently, multi-analyte detection requires multi-sensor arrays with individual specific coatings.

#### 3.3.1. QCM Bio- and Chemosensor Arrays

QCM biosensors have mainly been developed for medical applications. A 2 × 5 array of QCM sensors with antibody coatings was developed for monitoring the renal function by quantification of four nephropathy-related urinary proteins in urine samples. The limits of quantification determined for a coefficient of variation below 10% for five replicates were few to several µg/L [48]. The same setup was used for determining five pathogenic bacteria in wound secretion and pus. The pathogens were detected by means of the bacterial DNA; hence, corresponding single-stranded DNA probes were immobilized on the sensor surfaces. Measurable bacterial concentrations ranged from 1.5·10^2^ to 1.5·10^8^ CFU/mL (CFU: colony forming unit). The results regarding bacterial content being positive or negative were in good agreement with those from conventional culture techniques [36]. A 2 × 2 array of QCM sensors with coatings of leukemic lineage-associated CD (cluster of differentiation) antibodies was used for immunophenotyping of acute leukemia by detection of leukemia CD cell antigens in human bone marrow samples. The detection performance of the QCM immunoassay was comparable to immunohistochemistry, flow cytometry, and fluoroimmunoassay [183]. For the detection of drug residues in livestock production, an array of three MIP-coated QCMs was developed to detect clenbuterol and structural analogues of two of its metabolites in swine urine. The analytes could clearly be differentiated. The LOD for clenbuterol was determined to be 10 nM (3 ng/mL), which was comparable to other devices [184].

Newer developments of QCM biosensor arrays include a monolithic QCM sensor array with three sensing areas and a wireless, electrodeless QCM immunosensor array with up to ten channels. The performances of these arrays regarding multi-analyte detection were demonstrated by detection of multiple proteins using the corresponding antibody coatings or, in the case of antibody detection, with an additional protein A coating. The analyte proteins bound preferably on surfaces with the corresponding binding partners, while nonspecific binding was observed only to a small extent. As the research focused mainly on the functionality of the newly introduced array setups, comparatively high analyte concentrations were applied, ranging from several to several hundred µg/mL [34,105]. 

#### 3.3.2. FBAR Biosensor Arrays

The development of FBAR biosensor arrays for multi-analyte detection is just beginning. Arrays made of up to 64 FBARs were introduced for multiple protein detection by means of corresponding antibody coatings and for multiplexed DNA measurements. Two antibodies applied at a concentration of 1 µg/mL showed specific binding to the respective corresponding antibody coatings. DNA strands applied at a concentration of 1 µM in diluted serum (1:100) also bound only to the corresponding coatings while nonspecific binding from serum components was negligible [51,185]. 

#### 3.3.3. SAW Biosensor Arrays

SH-SAW dual delay-line devices based on a 42.5° rotated *Y*-cut, *z*-propagating quartz crystal were provided with PMMA layers to allow operation with Love waves. Each device was combined with a four-channel microfluidic setup in a way that, on each line, four subareas were created, i.e., eight subareas were obtained in total (µF-on-SAW; see Section 2.3). Phase shifts corresponding mainly to mass adsorption or desorption were used as signal response. Coating each line completely with a different receptor should allow that only the corresponding proteins would bind on the respective line. This was confirmed by selective detection of four biotinylated proteins (50 µg/mL) with neutravidin coatings and by selective antibody detection (50 µg/mL) with protein G coatings. Furthermore, specific protein detection (100 µg/mL) was obtained via coating the subareas with lipids containing different functional head groups. Coating the four subareas with four different antibodies enabled the specific detection of four corresponding cardiac markers, allowing cardiovascular risk assessment. Both established markers (creatine kinase-MB (CK-MB) and C-reactive protein (CRP)) and potential future heart disease markers (D-dimer and pregnancy-associated plasma protein A (PAPP-A)) were applied in concentrations ranging from 0.25–20 µg/mL in buffer, allowing the detection of critically high protein concentrations. However, the critical cutoff values could be detected only for CRP, with a cutoff concentration range of 1–10 µg/mL. The cutoff concentrations of the other cardiac markers are in the sub-µg/mL range and, hence, were too small for detection here. Furthermore, the measurements have not yet been performed with real serum samples [35,186,187]. 

## 4. Conclusions

Despite the many requirements to be met when working with acoustic sensors and sensor arrays, acoustic sensor arrays have successfully been utilized in numerous applications for multi-analyte detection in gaseous and liquid samples. In most cases, established BAW and SAW sensor devices and configurations have been used, with the research focusing on layer optimization and new applications. Newer sensor developments aim at the introduction of FBARs as sensor devices, which promise higher sensitivities because of the very high frequencies. However, for higher mass-sensitivities, the noise of these devices is still to be reduced. Furthermore, fabrication and signal recording of FBARs is not yet standardized, i.e., more investigations are required to get the desired low-cost, high-performance devices. Other sensor system developments aim at wireless or wireless and electrodeless readouts. Such configurations can increase the sensor performance, as shown for QCM devices. Furthermore, wireless sensor systems will allow monitoring of hazardous substances from a safe distance, particularly when increased to a network of multiple wireless sensor arrays.

Regarding device coatings, polymers have been and continue to be widely used, with further developments on newly developed polymers and imprinting processes. A newly introduced approach is the use of biomolecules (DNA, peptides, and proteins) in gas sensing applications to introduce new interaction mechanisms for better selectivity. First results are promising, and investigations of the stability will show in which way the new layers can compete with the conventional ones. However, particularly for newly introduced coatings, the layer stability often is not yet sufficiently investigated regarding storage or, in the case of chemical sensors, performance in multiple measurements.

Array development itself goes in two opposite directions. On the one hand, the number of sensors is increased (e.g., by using FBARs); on the other hand, the sensor number is reduced while the signal processing is enhanced, leading to virtual arrays. The latter has the advantage that, by using fewer sensors, the number of different sensor drifts interfering with the sensor signals is reduced, which should facilitate the calibration effort. However, particularly if the analytes to be detected are very similar, a certain number of sensors is still required to ensure selective detection of the individual components. Recent studies combine both approaches to obtain high-performance arrays with as little complexity as possible. 

While sensor arrays for chemical sensors and e-noses are very common, approaches for biosensor arrays are still inadequately represented, though their feasibility has been demonstrated. The reason for this may be that, for instance, biomarker profiles for diagnostic applications, which could easily be determined with such arrays, have not yet been fully identified, so that the key applications of these arrays are not yet fully defined.

## Figures and Tables

**Table 1:** Commercially available QCM multi-sensor arrays.

Company (Headquarter Location), URL ^1^	Model (s)	Channels	Fundamental Frequency (MHz)	Measurement Parameter (s)
**3T** (DE), www.3t-analytik.de	qCell series qCell T series	1, 2, or 4 1 or 2	n/a	Frequency and Dissipation
**Attana** (SE), www.attana.com	Attana 200/A200 Attana Cell 200/A200	2 2	n/a	Frequency
**AWSensors** (ES), https://awsensors.com	AWS A20+ RP	1–4 ^2^	5, 9, 10, 50, 100, 150	Frequency and Dissipation
**Biolin Scientific** (SE), www.biolinscientific.com	QSense Analyzer QSense Pro	4 8	5	Frequency and Dissipation
**Initium** (JP), www.initium2000.com	Affinix Q8/Qµ	8/1–4	27	Frequency
**MicroVacuum** (HU), https://microvacuum.com	QCM-I	2 or 4	5	Frequency and Dissipation
**Nihon Dempa Kogyo** (JP), www.ndk.com	NAPiCOS series	monolithic twin sensor	30	Frequency

^1^ Access date: 20 September 2019; ^2^ Array also available with Love wave SAW sensors (120 MHz).

**Table 2:** Commercially available e-noses based on acoustic sensors.

Company (Headquarter Location), URL ^1^	Model (s)	Type of Sensor (s)	Application
**Electronic Sensor Technology** (US), www.estcal.com	zNose series	GC-SAW (uncoated sensor)	Determination of a large variety of gas, VOCs ^2^, and vapor mixtures
**ENMET** (US), www.enmet.com	HAZMATCAD	SAW sensor array	Detection of CWAs ^3^ (Nerve and blister)
**Karlsruhe Institute of Technology** (DE), www.kit-technology.de	SAGAS	SAW sensor array (8 sensors)	Determination of gas mixtures
**Nihon Dempa Kogy**o (JP), www.ndk.com	Twin-CQCM, Twin-TQCM	Monolithic twin QCMs (1 to 4)	Outgas sensing
**Spectro Scientific** (US), www.spectrosci.com	FDM 6000	1 SAW sensor (polymer-coated)	Determination of fuel contaminants in lubricants

^1^ Access date: 23 September 2019; ^2^ volatile organic compounds; ^3^ chemical warfare agents.
